# Policies for care during the third stage of labour: a survey of maternity units in Syria

**DOI:** 10.1186/1471-2393-10-32

**Published:** 2010-06-22

**Authors:** Hosam E Matar, Muhammad Q Almerie, Mohamad Alsabbagh, Muhammad Jawoosh, Yara Almerie, Asma Abdulsalam, Lelia Duley

**Affiliations:** 1 North Middlesex University Hospital NHS Trust, Academic Foundation Trainee, Sterling Way, London N18 1QX, UK; 2 Princess of Wales Hospital, Foundation Trainee, Grimsby DN33 2BA, UK; 3 The Women's University Teaching Hospital, Faculty of Medicine, Damascus University, P.O Box: 14429, Damascus, Syria; 4 Fieldhouse Education Centre, Bradford Teaching Hospitals Foundation Trust, Bradford Royal Infirmary, Duckworth Lane Bradford, BD9 6RJ, UK

## Abstract

**Background:**

Care for women during the third stage aims to reduce the risk of major haemorrhage, but is very variable. The current World Health Organisation (WHO) recommendation is that care should include administration of a uterotonic (oxytocin, if it is available) soon after birth of the baby, delayed cord clamping, and delivery of the placenta by controlled cord traction.

**Methods:**

To ascertain care policies used during the third stage of labour in maternity units in Syria, we conducted a survey of 69 maternity units in obstetric and general public hospitals. A brief questionnaire was administered by face to face interview or telephone with senior obstetricians and midwives. Outcome measures were the use of prophylactic uterotonic drugs, timing of cord clamping, use of controlled cord traction, and treatment for postpartum haemorrhage. Obstetricians were asked about both vaginal and caesarean births, midwives only about vaginal births.

**Results:**

Responses were obtained for 66 (96%) hospitals: a midwife and an obstetrician were interviewed in 40; an obstetrician only in 20; a midwife only in 6. Responses were similar, although midwives were more likely to report that the umbilical cord was clamped after 1-3 minutes or after cessation of pulsation (2/40 obstetricians and 9/40 midwives). Responses have therefore been combined.

One hospital reported never using a prophylactic uterotonic drug. The uterotonic was Syntometrine^® ^(oxytocin and ergometrine) in two thirds of hospitals; given after delivery of the placenta in 60 (91%) for vaginal births, and in 47 (78%) for caesarean births. Cord clamping was within 20 seconds at 42 hospitals 64%) for vaginal births and 45 (75%) for caesarean births. Controlled cord traction was never used in a quarter (17/66) of hospitals for vaginal births and a half (32/60) for caesarean births.

68% of respondents (45/66) thought there was a need for more randomised trials of interventions during the third stage of labour.

**Conclusion:**

Most maternity units report using Syntometrine^®^, usually given after delivery of the placenta, clamping the cord within 20 seconds, and using controlled cord traction.

## Background

During the third stage of labour, which is from birth of the baby to delivery of the placenta, women may be at risk of major haemorrhage, particularly if the uterus does not retract adequately or the placenta partially separates. A quarter of the estimated 529,000 maternal deaths which occur each year, almost all in low and middle income countries [1], are associated with postpartum haemorrhage [2]. Postpartum haemorrhage also contributes to anaemia and other maternal morbidity following childbirth [3].

Care for women during the third stage aims to reduce the risk of major haemorrhage. Expectant, or physiological, care is based on awaiting physiological separation and delivery of the placenta, with intervention only if indicated. Active management is a package of care to accelerate retraction of the uterus, and separation and delivery of the placenta; thereby minimising blood loss [4]. Traditionally it includes administration of a prophylactic uterotonic drug, clamping and cutting of the umbilical cord, and controlled traction on the cord [5]. Prendiville *et al *in their Cochrane Systematic review, due to be updated, found that active management halves the risk of postpartum haemorrhage compared with expectant management [4]. Much of this reduction is due to the prophylactic uterotonic drug: [6,7] oxytocin is the drug of choice, as it has comparable impact on blood loss with fewer adverse effects than ergot alkoids [6]. The impact of other components, such as when is best to give the uterotonic [8], when to clamp the cord [9] and whether to use controlled cord traction [10], is unclear. Although there is no clear consensus about the components of active management of the third stage of labour [4,11], the current World Health Organisation (WHO) recommendation is that it should include administration of a uterotonic (oxytocin, if it is available) soon after birth of the baby, delayed cord clamping, and delivery of the placenta by controlled cord traction [12].

Developing effective strategies to prevent postpartum haemorrhage requires reliable evidence about the effects of interventions, and an understanding of current practice. Information about care during the third stage in low and middle income countries is scarce, however [13]. Syria is a lower middle income country with a population of 19.8 million people and annual growth of 2.5% [1,14]. The population is young (median age 21 years compared to 39 years in the UK) with high fertility (3.3 compared to 1.8 in the UK) [1]. Maternal mortality is 58 deaths per 100,000 live births [15], and 93% of women have a skilled birth attendant [15]. The caesarean section rate is 15%, compared to 23% in the UK [11,12]. In Syria the role of midwives includes providing antenatal and postnatal care, and conducting normal vaginal births in urban maternity units. Community midwives provide care for women in their own homes, as well as attending home births [11,16]. We surveyed hospitals providing maternity care in Syria to ask about current polices for care during the third stage of labour, and assessed how these compared to current evidence.

## Methods

All public hospitals run by the Ministry of Health, the Ministry of Higher Education (University Hospitals), and the Ministry of Defence (Military Hospitals) in Syria were identified through the Syrian National Statistics Centre. Around 30 small maternity units in remote areas and all private institutions were excluded, as they were difficult to contact and the number of births at each was small. The questionnaire was adapted from a self-completed questionnaire developed for use in the UK [17,18], and translated into Arabic. It was administered either by face to face interview or by telephone with a senior obstetrician at each hospital during 2008. Where possible, a senior midwife was also interviewed. It was explained that the survey asked about policy within the hospital, rather than the respondents' personal practice. The questionnaire was anonymous, and asked about use of prophylactic uterotonic drugs, timing of cord clamping, use of cord traction, and treatment of postpartum haemorrhage. The obstetricians' questionnaire asked about third stage care for both vaginal and caesarean births, the midwives' questionnaire only about vaginal births.

Data were entered into an excel spreadsheet and double checked. Analyses were performed using the Statistical Package for the Social Sciences (SPSS), version 13.0 (SPSS Inc., Chicago, IL, USA). The study protocol was approved by the Clinical Research Ethics Committee of the Faculty of Medicine at Damascus University and the Ministry of Health in Syria.

## Results

Sixty nine public hospitals were identified, of which nine were military hospitals. At least one response was obtained for 66 (96%) of hospitals: for 40 hospitals both a midwife and an obstetrician were interviewed, for 20 hospitals an obstetrician only and for six a midwife only. For the 40 hospitals where both an obstetrician and midwife responded, responses were similar between the two groups, although midwives were more likely to report that the umbilical cord was clamped after 1-3 minutes or after cessation of pulsation (2/40 obstetricians and 9/40 midwives). Responses are therefore combined, using the sixty obstetricians' responses plus the midwife responses for the six hospitals where only a midwife was interviewed.

One hospital reported never using a prophylactic uterotonic drug (Table 1). For two thirds of hospitals the choice of prophylactic uterotonic drug was Syntometrine^® ^(oxytocin and ergometrine); this was given after delivery of the placenta in 91% (60/66 hospitals) for vaginal births and in 78% (47/60) for caesarean births (Table 1). Cord clamping was within 20 seconds at 42 hospitals 64%) for vaginal births and 45 (75%) for caesarean births. Draining the placenta after cord clamping was never used in 74% (49/66) for vaginal births and 87% (52/60) for Caesarean births. Controlled cord traction was never used in a quarter (17/66) of the hospitals for vaginal births and more than half (32/60) for caesarean births.

**Table 1:** Polices for care during the third stage of labour for vaginal and caesarean births†

	Vaginal births n = 66 (%)		Caesarean births n = 60 (%)	
***Use of a prophylactic uterotonic drug***				
*How often is a prophylactic uterotonic used?*				
always/usually	61	(92)	56	(93)
sometimes	4	(6)	3	(5)
Rarely	-	-	-	-
Never	1	(2)	1	(2)
				
Which uterotonic is usually used?*				
Syntometrine‡	45	(68)	40	(67)
ergometrine	13	(20)	8	(13)
oxytocin	7	(11)	9	(15)
Syntometrine‡ + misoprostol	-	-	2	(3)
				
When is the uterotonic drug usually administered?*				
with the anterior shoulder	-	-	-	-
with delivery of the body	4	(6)	1	(2)
after birth of baby, before cord clamping	1	(2)	-	-
after birth of baby, after cord clamping	-	-	11	(18)
after delivery of the placenta	60	(91)	47	(78)
				
***Umbilical cord clamping***				
*When is the cord usually clamped?*				
within 20 seconds	42	(64)	45	(75)
after 20 seconds - 1 minute	20	(30)	13	(22)
after 1-3 minutes	2	(3)	1	(2)
after >3 minutes	1	(2)	-	-
after cessation of pulsation	1	(2)	1	(2)
				
*After cutting the cord, is the clamp released to drain the placenta?*				
always/usually	4	(6)	1	(2)
sometimes	7	(11)	3	(5)
Rarely	6	(9)	4	(7)
Never	49	(74)	52	(87)
				
***Is controlled cord traction used?***				
always/usually	29	(44)	18	(30)
sometimes	14	(21)	8	(13)
Rarely	6	(9)	2	(3)
never	17	(26)	32	(53)

† Results presented combined for obstetricians and midwives within the practice

‡ oxytocin plus ergometrine

* not applicable for one hospital where a prophylactic uterotonic is never used

Two thirds (68%, 45/66) of those interviewed thought more research evidence from randomised trials of interventions during third stage was needed. The questions they thought important were when to give the prophylactic uterotonic (82%, 37/45), when to clamp the cord (29%, 13/45) and whether to use controlled cord traction, or not (20%, 9/45).

For treatment of postpartum haemorrhage, 80% (53/66) of hospitals reported always or usually using uterine massage; a third (33%, 22/66) always or usually catheterise the bladder; a quarter (23%, 15/66) always or usually use bimanual compression of the uterus, and two thirds (61%, 40/66) use Syntometrine^® ^as the drug of first choice (Table 2).

**Table 2:** Immediate treatment for postpartum haemorrhage

	n = 66 (%)
*Is uterine massage used?*	
always/usually	53 (80)
sometimes	10 (15)
rarely	3 (5)
never	-
	
*Is the bladder catheterised?*	
always/usually	22 (33)
sometimes	21 (39)
rarely	14 (21)
never	9 (14)
	
*Is bimanual compression of the uterus used?*	
always/usually	15 (23)
sometimes	21 (32)
rarely	15 (23)
never	15 (23)
	
*What is the drug of first choice for treatment of postpartum haemorrhage?*	
Syntometrine^†^	40 (61)
ergometrine alone	10 (15)
oxytocin alone	8 (12)
Syntometrine^† ^+ misoprostol	6 (9)
misoprostol alone	2 (3)

^† ^oxytocin and ergometrine

## Discussion

Our survey of maternity unit policy for care during the third stage of labour in Syria found poor compliance with WHO recommendations [19]. Although use of a prophylactic uterotonic drug was almost universal, and has improved since 2000 when only 13% of Syrian hospitals reported using a uterotonic for normal births [16], the choice of drug was usually Syntometrine^®^. The uterotonic drug was usually given after delivery of the placenta. Optimal timing for administration of the uterotonic is unclear. The WHO recommendation that this be 'soon after birth of the baby' [19] implies before delivery of the placenta; and to be considered active management of the third stage it should be given during the third stage. The uterotonic drug is given after delivery of the placenta in some European countries [20], and in the USA [21].

Timing of cord clamping is controversial. Although WHO recommends delayed clamping, how long to delay is not specified [19]. The Cochrane Review shows promising benefits associated with delayed, or deferred, clamping rather than early clamping, but further evidence is needed to provide evidence about long term outcome and reassurance about safety [9]. The relatively low use of controlled cord traction is similar to many countries in Europe [20], although not to the UK where this practice is widespread [18,20].

Our study only included public hospitals. However, it is in public hospitals where most people in Syria receive their care, and it is in these that most of the morbidity and mortality occurs. Also, we did not observe actual practice which may have differed from reported policy. Midwives' and obstetricians' responses were similar within each unit; however, suggesting policy may have been agreed within the hospital.

We did not explore reasons why reported policies for active management of the third stage did not comply with current international recommendations. However, an earlier study in Egypt found that factors were lack of written protocols, particularly for normal births, poor understanding of active and physiological management, and fear that early use of a prophylactic uterotonic might trap the placenta in the uterus [22]. This belief that early uterotonic use will lead to retained placenta is also reported in the USA [21]. For women with low risk of haemorrhage, active management is associated with an increase in the need for manual removal of the placenta, compared with physiological management [4]. Further research is needed to assess whether this risk is influenced by timing of administration of the uterotonic, or by reducing the placental circulation, either by deferred cord clamping or placental drainage.

Variations in strategies used for treatment of women with postpartum haemorrhage were also reported. Similar variation has been reported elsewhere, and there is a need for better evidence to support guidelines for treatment of women with postpartum haemorrhage [20,23]. Advanced life support in obstetrics [24] in addition to the traditional methods such as massaging the uterus, bladder catheterisation, bimanual compression and the use of a uterotonic drug have been implemented in Syria, although there are no national guidelines [16]. Our findings suggest a national policy for postpartum haemorrhage would help provide more coherent care in Syria.

## Conclusions

Most maternity units report using Syntometrine^®^, usually given after delivery of the placenta, clamping the cord within 20 seconds, and using controlled cord traction. Although active management of the third stage of labour is widely recommended to reduce the risk of postpartum haemorrhage, there remains uncertainty about the effects of some of the components. Nevertheless, this survey suggests that there is scope for improving compliance with evidence-based practice for care during the third stage of labour in Syria.

## Competing interests

The authors declare that they have no competing interests.

## Authors' contributions

LD conceived the idea for the study and is guarantor. HEM, LD, MQA and AA contributed to design of the study. YA, MJ and MA managed organisation and data collection. HEM, MQA and LD did the analysis and drafted the report. All authors have commented on the report and approved the final version.

## Pre-publication history

The pre-publication history for this paper can be accessed here:

http://www.biomedcentral.com/1471-2393/10/32/prepub

## References

[B1] The World Health Report 2005 Make every mother and child count 2005 Geneva. World Health Organization 10.1080/14034940500217037 16332605

[B2] Khan KS Wojdyla D Say L Gulmezoglu AM Van Look PF WHO analysis of causes of maternal death: a systematic review Lancet 2006 367 1066 1074 10.1016/S0140-6736(06)68397-9 16581405

[B3] AbouZahr C Global burden of maternal death and disability British Medical Buletin 2003 67 1 11 10.1093/bmb/ldg015 14711750

[B4] Prendiville WJ Elbourne D McDonald S Active versus expectant management in the third stage of labour Cochrane Database Syst Rev 2000 CD000007 10.1002/14651858.CD000007 10796082

[B5] Brucker MC Management of the third stage of labor: an evidence-based approach Journal of Midwifery and Women's Health 2001 46 381 392 10.1016/S1526-9523(01)00192-1 11783686

[B6] Cotter A Ness A Tolosa J Prophylactic use of oxytocin in the third stage of labour Cochrane Database Syst Rev 2001 CD001808 24173606 10.1002/14651858.CD001808.pub2

[B7] Liabsuetrakul T Choobun T Peeyananjarassri K Islam QM Prophylactic use of ergot alkaloids in the third stage of labour Cochrane Database Syst Rev 2007 CD005456 17443592 10.1002/14651858.CD005456.pub2

[B8] Soltani H Dickinson F Timing of prophylactic oxytocics for the third stage of labour after vaginal birth Cochrane Database Syst Rev 2006 CD004665 10.1002/14651858.CD006173.pub2 20687079

[B9] McDonald SJ Middleton P Effect of timing of umbilical cord clamping of term infants on maternal and neonatal outcomes Cochrane Database Syst Rev 2008 CD004074 18425897 10.1002/14651858.CD004074.pub2

[B10] Mshweshwe NT Hofmeyr GJ Gülmezoglu AM Controlled cord traction for the third stage of labour (Protocol) Cochrane Database of Systematic Reviews 2009 CD008020 10.1002/14651858.CD008020.pub2 PMC6464177 25631379

[B11] Family health survey in the Syrian Arab Republic summary report Syrian Arab Republic and the Pan Arab Project for Family Health (PAPFAM) of the League of Arab States: Central Bureau of Statistics [Syria] 2002

[B12] Jurdi R Khawaja M Caesarean section rates in the Arab region: a cross-national study Health Policy Plan 2004 19 101 110 10.1093/heapol/czh012 14982888 PMC1457106

[B13] Festin MR Lumbiganon P Tolosa JE Finney KA Ba-Thike K Chipato T Gaitan H Xu L Limpongsanurak S Mittal S International survey on variations in practice of the management of the third stage of labour Bul World Health Organ 2003 81 286 291 PMC2572442 12764495

[B14] Syrian Central Bureau of Statistics http://www.cbssyr.org/

[B15] Syrian Arab Republic, Minstry of Health http://www.moh.gov.sy/en/Statistics/HealthIndicators/tabid/337/portalid/0/Default.aspx

[B16] Abdulsalam A Bshour H Cheikha S Al-Faysal W Gabr A Al-Jorf S Khadra M Mashkouk S Wannous S Routine care of normal deliveries as applied in Syrian maternity wards Journal of the Arab Board of Medical Specializations 2004 6 134 140

[B17] Airey R Farrar D Duley L Timing of umbilical cord clamping: midwives' views and practice British Journal of Midwifery 2008 16 236 239

[B18] Farrar D Tuffnell D Airey R Howarth H Duley L Care during the third stage of labour: A postal survey of obstetricians and midwives in the UK Archives of Disease in Childhood - Fetal and Neonatal Edition 2009 94 Fa40 Fa57

[B19] WHO Recommendations for the Prevention of Postpartum Hemorrhage World Health Organisation. Department of Making Pregnancy Safer 2007

[B20] Winter C Macfarlane A Deneux-Tharaux C Zhang WH Alexander S Brocklehurst P Bouvier-Colle MH Prendiville W Cararach V van Roosmalen J Variations in policies for management of the third stage of labour and the immediate management of postpartum haemorrhage in Europe BJOG 2007 114 845 854 10.1111/j.1471-0528.2007.01377.x 17567419 PMC1974828

[B21] Jackson KW Allbert JR Schemmer GK Elliot M Humphrey A Taylor J A randomized controlled trial comparing oxytocin administration before and after placental delivery in the prevention of postpartum hemorrhage American Journal of Obstetrics and Gynecology 2001 185 873 877 10.1067/mob.2001.117363 11641669

[B22] Cherine M Khalil K Hassanein N Sholkamy H Breebaart M Elnoury A Management of the third stage of labor in an Egyption teaching hospital International Journal of Gynecology and Obstetrics 2004 87 54 58 10.1016/j.ijgo.2004.05.013 15464784 PMC1457103

[B23] Mousa HA Alfirevic Z Major postpartum hemorrhage: survey of maternity units in the United Kingdom Acta Obstet Gynecol Scand 2002 81 727 730 12174156 10.1034/j.1600-0412.2002.810807.x

[B24] Deutchman M Dresang L Winslow D Advanced life support in obstetrics (ALSO) international development Family Medicine 2007 39 618 622 17932793

